# Mid-treatment changes in intra-tumoural metabolic heterogeneity correlate to outcomes in oropharyngeal squamous cell carcinoma patients

**DOI:** 10.1186/s13550-025-01226-6

**Published:** 2025-04-01

**Authors:** Yuvnik Trada, Mark T. Lee, Michael G. Jameson, Phillip Chlap, Paul Keall, Daniel Moses, Peter Lin, Allan Fowler

**Affiliations:** 1 Department of Radiation Oncology, Calvary Mater Newcastle, Edith St, Waratah, 2298 NSW Australia; 2 Faculty of Medicine and Health, Sydney Medical School, The University of Sydney, Sydney, NSW Australia; 3 Department of Radiation Oncology, Cancer Therapy Centre, Liverpool Hospital, Liverpool, NSW Australia; 4 South Western Clinical School, School of Medicine, University of New South Wales, Sydney, NSW Australia; 5 GenesisCare, Sydney, NSW Australia; 6 St Vincent’s clinical school, Faculty of Medicine, University NSW, Sydney, Australia; 7 Ingham Institute of Applied Medical Research, Liverpool, NSW Australia; 8 Image X Institute, University of Sydney, Sydney, NSW Australia; 9 Graduate school of Biomedical Engineering, Faculty of Engineering, University of New South Wales, Sydney, Australia; 10 Department of Medical Imaging, Prince of Wales Hospital, Randwick, NSW Australia; 11 Department of Nuclear Medicine and PET, Liverpool Hospital, Liverpool, NSW Australia; 12 School of Medicine, Western Sydney University, Sydney, NSW Australia

**Keywords:** FDG-PET, Mid-treatment, Prognostic marker, Head and neck neoplasm, Oropharynx, Radiotherapy

## Abstract

**Background:**

This study evaluated mid-treatment changes in intra-tumoural metabolic heterogeneity and quantitative FDG-PET/CT imaging parameters and correlated the changes with treatment outcomes in oropharyngeal squamous cell cancer (OPSCC) patients. 114 patients from two independent cohorts underwent baseline and mid-treatment (week 3) FDG-PET. Standardized uptake value maximum (SUV_max_), standardized uptake value mean (SUV_mean_), metabolic tumour volume (MTV), and total lesional glycolysis (TLG) were measured. Intra-tumoural metabolic heterogeneity was quantified as the area under a cumulative SUV-volume histogram curve (AUC-CSH). Baseline and relative change (%∆) in imaging features were correlated to locoregional recurrence free survival (LRRFS) using multivariate Cox regression analysis. Patients were stratified into three risk groups utilising ∆AUC-CSH and known prognostic features, then compared using Kaplan-Meier analysis.

**Results:**

Median follow up was 39 months. 18% of patients developed locoregional recurrence at 2 years. A decrease in heterogeneity (∆AUC-CSH: 24%) was observed mid-treatment. There was no statistically significant difference in tumour heterogeneity (AUC-CSH) at baseline (*p* = 0.134) and change at week 3 (*p* = 0.306) between p16 positive and p16 negative patients. Baseline imaging features did not correlate to LRRFS. However, ∆MTV (aHR 1.04; 95% CI 1.03–1.06; *p* < 0.001) and ∆AUC-CSH (aHR 0.96; 95% CI 0.94–0.98; *p* = 0.004) were correlated to LRRFS. Stratification using ∆AUC-CSH and p16 status into three groups showed significant differences in LRR (2 year LRRFS 94%, 79%, 17%; log rank *p* < 0.001). Stratification using ∆AUC-CSH and ∆MTV into three groups showed significant differences in LRR (2 year LRRFS 93%, 70%, 17%; log rank *p* < 0.001).

**Conclusion:**

Mid-treatment changes in intra-tumoural FDG-PET/CT heterogeneity correlated with treatment outcomes in OPSCC and may help with response prediction. These findings suggest potential utility in designing future risk adaptive clinical trials.

**Supplementary Information:**

The online version contains supplementary material available at 10.1186/s13550-025-01226-6.

## Background

Definitive chemo/radiotherapy represents an established treatment approach for patients diagnosed with oropharyngeal squamous cell carcinoma (OPSCC). Nonetheless, a subset of these patients experience locoregional tumour recurrence following treatment, resulting in significant morbidity and mortality. In patients with controlled disease, treatment is associated with significant toxicities. Clinical features such as HPV status, tumour stage (TNM), and smoking history provide prognostic information but have failed to be useful as predictive markers in risk-adapted studies [1–4]. Therefore, there is a need for a reliable quantitative biomarker that can better risk stratify to allow tailored risk adapted intensification or de-intensification of treatment.

Functional imaging offers the advantage of non-invasively assessing biological characteristics of the entire tumour as opposed to tissue biopsies. Additionally, repeated imaging can also allow serial monitoring of the tumour during treatment that can act as early surrogate imaging biomarkers for treatment response. 2-[¹⁸F]fluoro-2-deoxy-D-glucose-positron emission tomography/ computed tomography (FDG-PET/CT) has the ability to assess tumour cellularity and metabolism, while also serving as an indirect measure of proliferation and hypoxia, factors strongly associated with radioresistance [5–7]. FDG-PET-derived first order features such as SUV_max_, SUV_mean_, MTV and TLG have been used to predict treatment outcomes in OPSCC patients with varying success [8]. Changes in imaging features during treatment have shown to be better predictors for treatment response compared to static baseline measures [8, 9]. Among these, changes in metabolic volumetric parameters such as MTV, show the greatest promise in OPSCC [10–12]. However, first-order volumetric parameters have limitations, including the failure to capture regional variations within the tumour and potential inaccuracies due to the inclusion of surrounding normal tissue with FDG uptake or treatment-related inflammation.

Cancers are also characterised by heterogeneity. Intra-tumour heterogeneity has been strongly associated with treatment resistance and represents a potentially novel imaging biomarker [13, 14]. Heterogeneity promotes tumour aggressiveness and therapeutic resistance and represents a significant challenge in the design of effective treatment strategies [14]. A histogram-based measure, such as AUC-CSH, when applied to FDG-PET imaging has the ability to provide a reproducible, reliable and easy to interpret quantitative measure of tumour heterogeneity [15, 16]. Tumour heterogeneity measured using AUC-CSH has been correlated to treatment outcomes on pre-treatment imaging in numerous malignancies including OPSCC [17–20]. There is currently limited evidence on change in tumour heterogeneity that occurs early during radiotherapy and how it may impact eventual treatment response.

The hypothesis for this study was that change in tumour heterogeneity correlates to treatment outcomes and can help risk stratify patients for individual risk-adapted treatment in oropharyngeal patients. The objectives of the study were to:

1. Measure changes in imaging features, including tumour heterogeneity, early during radiotherapy.
2. Compare the change in imaging features between HPV-associated and non-HPV-associated OPSCC.
3. Correlate baseline and mid-treatment FDG-PET imaging features, including tumour heterogeneity, to clinical outcomes and risk stratify patients.

## Methods

### Patient selection and treatment

Patients with newly diagnosed, biopsy-proven, non-metastatic oropharyngeal squamous cell carcinoma treated with definitive radiotherapy with or without concurrent systemic therapy from two independent cohorts treated at two large tertiary institutions were evaluated. Patients in Cohort One were retrospectively selected from a prospectively maintained database of patients who received treatment between May 2010 to October 2019. Patients in Cohort Two were recruited on a prospective quantitative imaging biomarker study from June 2014 to October 2019 [21]. Ethics approval was provided for both cohorts. Patient details gathered included age, ECOG, smoking, alcohol intake and gender. Tumour details gathered included TNM (AJCC 7th edition), T stage, *N* stage, tumour grade and primary site. HPV association was assessed by p16 immunohistochemistry staining, assessed as ≥ 70% tumour cells showing nuclear and cytoplasmic staining as per routine clinical practice.

All patients were evaluated and reviewed by a multidisciplinary team consisting of radiation oncologists, medical oncologists, surgeons and radiologists. Patients received treatment to three clinical target volumes (CTVs), delineated according to the latest consensus international guidelines utilising 5 + 5 mm expansion and elective nodal irradiation based on the primary tumour [22, 23]. Departmental guidelines included rigorous peer review, multidisciplinary plan review and patient specific quality assurance. Patients were treated using an IMRT/VMAT simultaneous integrated boost technique using daily image guidance and delivered using a linear accelerator with 6MV photons. Treating clinicians were blinded to the mid-treatment imaging results to minimise the risk of bias.

Patients in both cohorts underwent a baseline [18 F]FDG-PET before and during (week 3) radiotherapy. Treatment response was evaluated with a 3-month post-treatment FDG-PET and via regular clinical examinations including nasoendoscopy. Repeat imaging in follow-up were performed based on clinical suspicion or symptoms as per routine clinical practice. Recurrences were defined from treatment completion and confirmed histologically or via imaging following consensus discussion at a multidisciplinary head and neck meeting.

Primary outcome was locoregional recurrence free survival measured from time of diagnosis. Patients with distant metastatic recurrence only were followed for locoregional outcomes until death or initiation of systemic therapy.

### PET/CT scan parameters

FDG-PET studies were acquired in radiotherapy treatment position on a GE Discovery^TM^-710 PET-CT time-of-flight positron emission tomography (PET)-CT (GE Healthcare, Waukesha, MI). All scans were performed on the same scanner with the same acquisition and reconstruction protocols. The uptake time of the mid-treatment study was kept within 10% of the uptake time of the baseline study to ensure standardisation. Details of the imaging technique have been described previously [24].

### PET/CT image analysis

The region of interest (ROI) was the primary tumour. The tumour was volumetrically delineated using the PETedge tool of MIM software (MIM Software Inc, OH, USA), a semi-automated gradient method by a radiation oncologist in consensus with a nuclear medicine physician, both of whom were blinded to patients outcomes. PETEdge method was used as described previously by Werner-Waskit et al. and shown to closely approximate manual segmentation and pathological tumour volume [25–27]. Anatomical tumour volume on CT were not correlated with clinical outcomes based on our previous study results [24].

DICOM images were subsequently analysed using open-source PyRadiomics software (v2.2.0) [28]. Grey value discretisation was performed using a fixed bin size of SUV 0.3 with nil additional normalisation or post-processing. Fixed bin method was chosen due to greater reproducibility, and a size of SUV 0.3 was chosen to provide a reasonable number of bins for analysis [29]. FDG-PET imaging parameters including maximum SUV uptake (SUV_max_), mean SUV uptake (SUV_mean_), metabolic tumour volume (MTV) and total lesion glycolysis (TLG = SUV_mean_ × MTV) were calculated. Intra-tumoural metabolic heterogeneity was quantitatively calculated as the area under the curve of the cumulative SUV-volume histogram (AUC-CSH) obtained by plotting the percentage volume greater than the percentage of SUV_max_. Lower AUC-CSH value corresponding to higher degree of heterogeneity and value of 1 corresponding to perfect homogeneity [15]. Thus, a decrease in tumour heterogeneity during treatment would correspond with an increase in AUC-CSH value.

Five parameters were extracted for all ROIs at both time points. Percentage change (∆) in FDG-PET imaging parameters from baseline was calculated, defined as ∆ = (week 3– week 0)/week 0 × 100%.

A pre-specified cut-off value of ∆MTV was chosen as ≥ 50% change at week 3 as favourable imaging response based on previous study for exploratory analysis [10]. Given the lack of previous data on ∆AUC-CSH, we chose a pre-specified cut-off of > 0% defined as a positive change in tumour heterogeneity as favourable imaging response for further explorative analysis.

### Statistical analysis

The changes in FDG-PET parameters from baseline to week 3 were compared using the Wilcoxon signed-rank test. The correlation between absolute and changes (∆) in imaging features were estimated using Spearman’s Rho test to identify redundant features with very strong correlation (Rs > 0.80). Correlations were defined as very strong (1.00-0.81), strong (0.80 − 0.61), moderate (0.60 − 0.41), weak (0.40–0.51) and negligible (0.20-0.00) [30]. Differences in clinical and FDG-PET imaging features were correlated to p16 status using Mann-Whitney U test.

Univariate and multivariate Cox regression was utilised to correlate clinical and imaging features to locoregional recurrence. A-priori selected features (p16 status, TNM stage, chemotherapy) with previously well demonstrated prognostic value were utilised for multivariate analysis [31, 32].

#### Risk stratification using tumour heterogeneity

To determine the utility of using tumour heterogeneity for risk stratification, two analysis methods were undertaken. First risk stratification method combined pre-specified ∆AUC-CSH (positive change vs. negative change) and p16 status into three groups (p16 negative; p16 positive with unfavourable ∆AUC-CSH; and p16 positive with favourable ∆AUC-CSH). Second risk stratification method utilised change in metabolic tumour volume (∆MTV) with previously demonstrated prognostic ability in OPSCC [10, 24, 33]. It combined change in tumour volume with tumour heterogeneity using pre-specified cut-off values of ∆MTV and ∆AUC-CSH into three groups; favourable ∆MTV, unfavourable ∆MTV and favourable ∆AUC-CSH, or unfavourable ∆MTV and unfavourable ∆AUC-CSH. Kaplan-Meier survival analysis and Mantle-Cox log-rank test were performed to compare the groups. Rates of disease control at 2 years (2 year LRRFS) were presented, a clinically significant time-point given that LRR recurrences after this period are uncommon.

The data were analysed using SPSS statistical software (Version 24.0; IBM Corp, Armonk, NY, USA). Statistical significance was considered as *p* < 0.05.

## Results

### Patient characteristics

114 patients were included in the study, 78 patients in Cohort One and 36 in Cohort Two. Patient and tumour characteristics details are summarised in Table 1. There were no statistical differences in patient characteristics between the two cohorts. The majority of patients (85%) were treated with concurrent chemoradiotherapy. The median follow up was 39.1 months (range 5–88 months). At two years of follow-up, 21 patients (18%) experienced locoregional recurrence and 19 patients (17%) had died. Among those with locoregional recurrence, 9 patients (43%) had local recurrence only, 7 patients (33%) had nodal recurrence only and 5 patients (24%) had both local and nodal recurrence.

**Table 1:** Patient characteristics

		Combined (*n* = 114)	Cohort 1 (*n* = 78)	Cohort 2 (*n* = 36)	*P* value
Age at diagnosis (years)		62.0 ± 9.6	62.1 ± 9.3	61.7 ± 10.4	0.494
Gender	Male	97 (85%)	65 (83%)	32 (89%)	0.439
	Female	17 (15%)	13 (17%)	4 (11%)	
Performance status	ECOG 0	63 (55%)	40 (51%)	23 (64%)	0.441
	ECOG 1	42 (37%)	31 (40%)	11 (31%)	
	ECOG 2	9 (8%)	7 (9%)	2 (6%)	
Smoker	No	30 (26%)	20 (26%)	10 (28%)	0.822*
	Yes	84 (74%)	58 (74%)	26 (72%)	
Smoking history (pack/yr)		26.9 ± 24.4	27.1 ± 24.5	26.4 ± 24.6	0.895
Alcohol intake	Nil	24 (21%)	18 (23%)	6 (17%)	0.417
	< 1 SD/day	28 (25%)	16 (20%)	12 (33%)	
	1–3 SD/day	20 (18%)	15 (19%)	5 (14%)	
	> 3 SD/day	25 (22%)	19 (24%)	6 (17%)	
	Ex-heavy (3 SD/day)	17 (15%)	10 (13%)	7 (19%)	
Primary tumour site	Tonsil	51 (45%)	33 (42%)	18 (50%)	0.757
	Base of tongue	45 (39%)	31 (40%)	14 (39%)	
	Soft palate	10 (9%)	8 (10%)	2 (6%)	
	PPW	6 (5%)	4 (5%)	2 (6%)	
	UNPSCC	2 (2%)	2 (3%)	0 (0%)	
TNM stage	Stage 2	6 (5%)	4 (5%)	2 (6%)	0.921
	Stage 3	18 (16%)	13 (17%)	5 (14%)	
	Stage 4a	77 (68%)	51 (65%)	26 (72%)	
	Stage 4b	10 (9%)	8 (10%)	2 (6%)	
	Stage 4c	3 (3%)	2 (3%)	1 (3%)	
T stage	T0	3 (3%)	3 (4%)	0 (0%)	0.200
	T1	5 (4%)	4 (5%)	16 (44%)	
	T2	39 (34%)	23 (30%)	15 (42%)	
	T3	43 (38%)	28 (36%)	15 (42%)	
	T4	24 (21%)	20 (26%)	4 (11%)	
Grade	Well differentiated	12 (11%)	11 (14%)	1 (3%)	0.176
	Mod differentiated	17 (15%)	13 (17%)	4 (11%)	
	Poor differentiated	37 (33%)	25 (32%)	12 (33%)	
	Unknown	48 (42%)	29 (37%)	19 (53%)	
P16 status	Negative	28 (25%)	22 (28%)	6 (17%)	0.350
	Positive	55 (48%)	37 (47%)	18 (50%)	
	Unknown/NA	31 (27%)	19 (24%)	12 (33%)	
Treatment	Radiotherapy alone	17 (15%)	13 (17%)	4 (11%)	0.576*
	Chemoradiotherapy	97 (85%)	65 (83%)	32 (89%)	
Median follow-up (months)		39.1	35.3	34.7	0.643

Continuous variables are presented in mean ± standard deviation and compared using Mann-Whitney U test. Categorical data are presented as numbers (%) and compared using Chi-square (x^2^) test or Fisher’s Exact test*

### Change in imaging features during radiotherapy

**Fig. 1:**
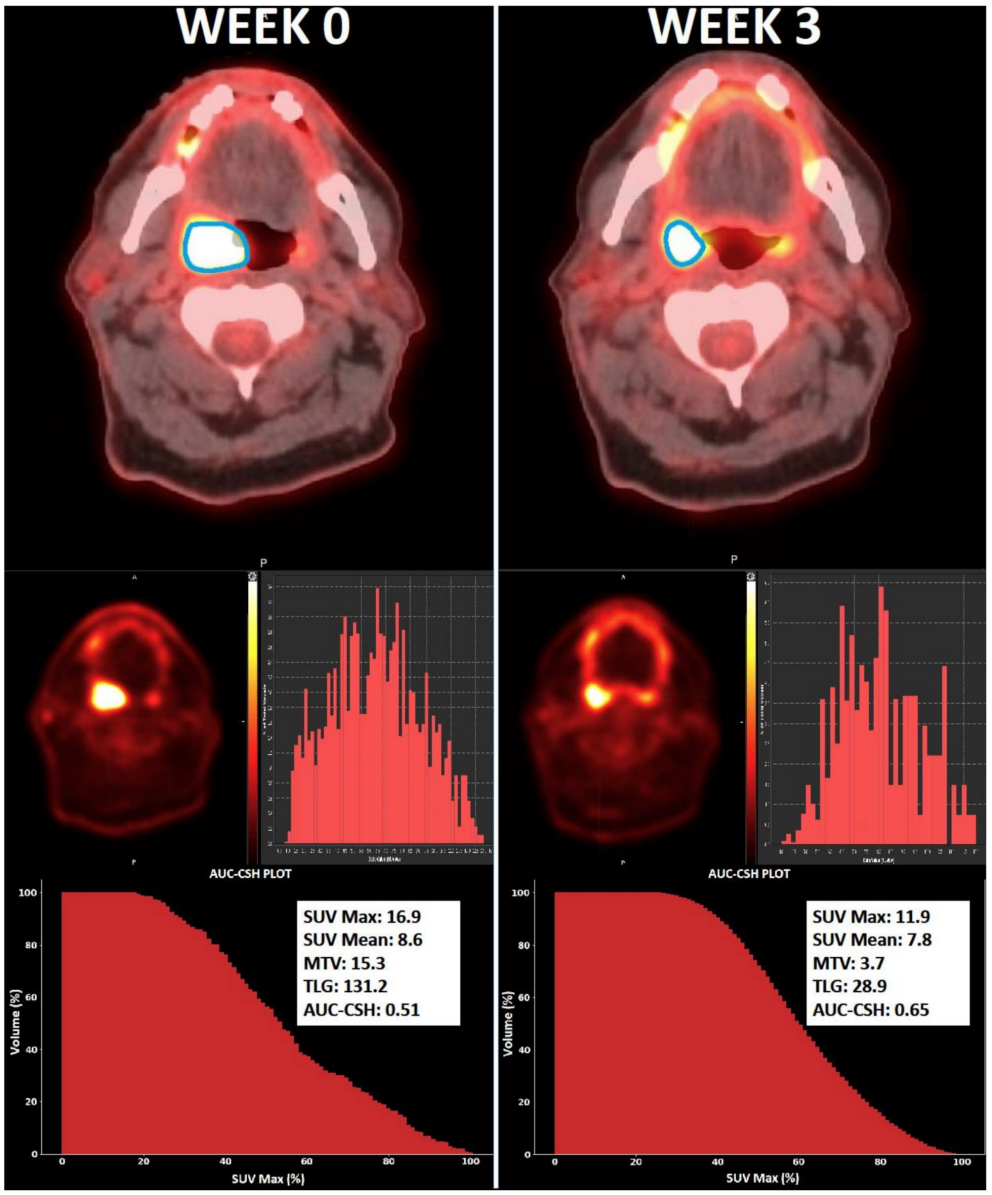
Case example of a patient with stage IVa HPV-positive tonsil squamous cell carcinoma (T2N1M0) who exhibited a favourable mid-treatment FDG-PET imaging response after chemo-radiotherapy and remained disease-free 5 years post-diagnosis

Statistically significant decreases in SUVmax, SUVmean, MTV and TLG were noted at week 3 compared to baseline (*p* < 0.001), see supplementary Table 1. On average there was a larger reduction in MTV (-50%) and TLG (-63%) at week 3 from baseline, as compared to the reduction in SUVmax (-37%) and SUVmean (-25%). There was a statistically significant reduction in tumour heterogeneity (AUC-CSH) at week 3 compared to baseline (*p* < 0.001). On average the tumour heterogeneity reduced by 24% at week 3 during radiotherapy. An example of a patient with good AUC-CSH change during treatment is provided in Fig. 1. There were only weak or moderate correlations between AUC-CSH and the other imaging features (Rs < 0.57). See supplementary Fig. 1 and Fig.  for scatterplot of correlation of AUC-CSH with other imaging features.

**Fig. 2:**
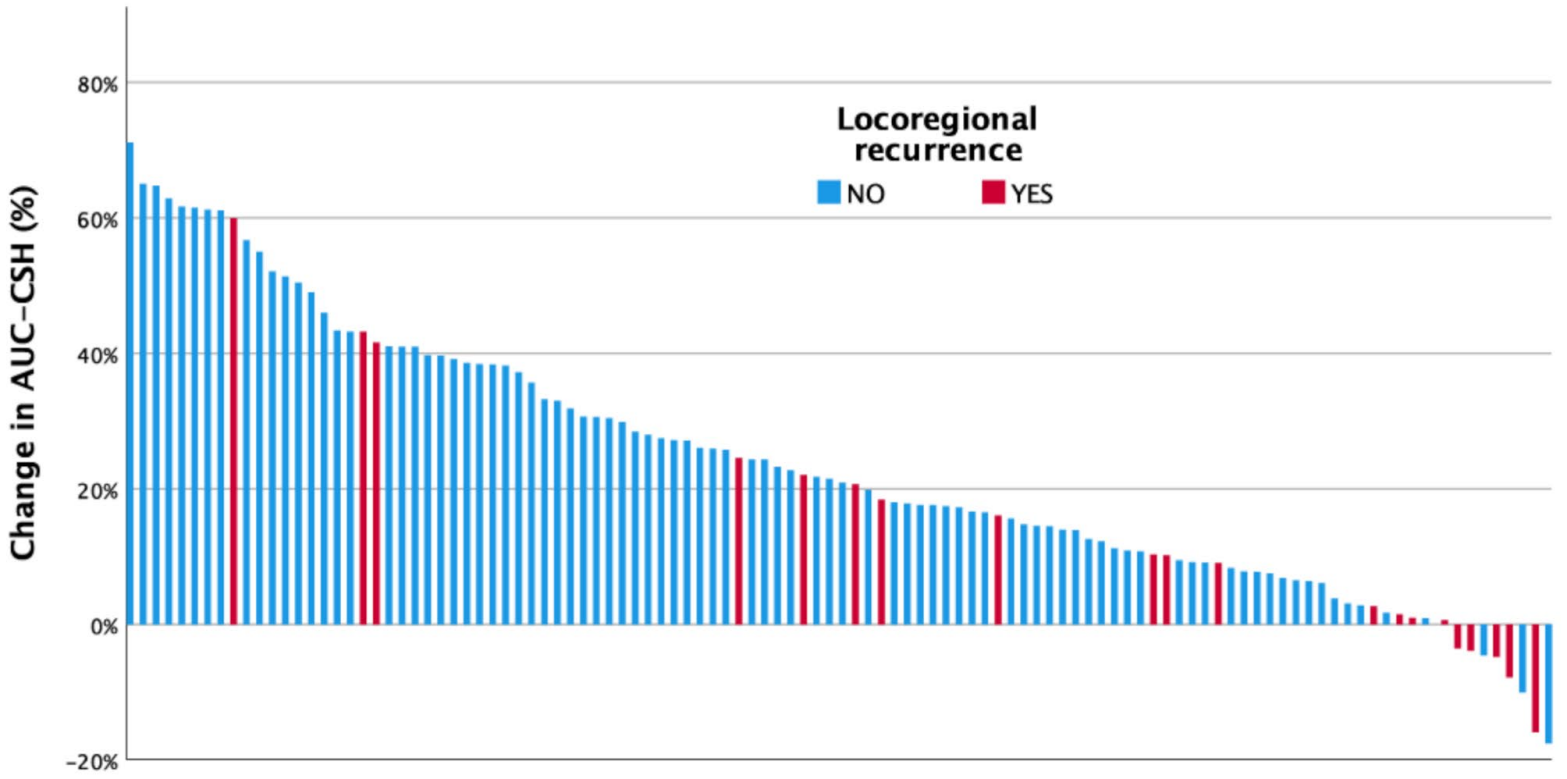
Waterfall plot of percentage change in AUC-CSH at week 3 during radiotherapy, patients with locoregional recurrence (red) and without locoregional recurrence (blue)

### Comparison of clinical and imaging features by p16 status

Comparison between patients with HPV associated (p16 positive) and non-HPV-associated (p16 negative) OPSCC are described in supplementary Table 3. When comparing clinical features, patients with p16 positive disease had lower ECOG status, smoking history and alcohol intake compared to p16 negative patients. When comparing imaging features, only baseline primary tumour SUV_mean_ was statistically different between p16 positive and p16 negative patients (SUV 6.8 vs. 5.9, *p* = 0.028), see Table 2. There was no statistically significant difference in tumour heterogeneity (AUC-CSH) at baseline (*p* = 0.134) and change at week 3 (*p* = 0.306) between p16 positive and p16 negative patients.

**Table 2:** Comparison of imaging parameters between patients by p16 status

PARAMETER	P16 negative (mean, SD)	P16 positive (mean, SD)	*P* value*
**FDG-PET IMAGING FEATURES**			
**Week 0**			
SUV_max_	12.6, 5.2	13.5, 4.7	0.469
SUV_mean_	5.9, 2.0	6.8, 2.1	**0.028**
MTV	25.2, 25.0	21.5, 23.9	0.694
TLG	169.8, 187.4	153.2, 179.5	0.885
AUC-CSH	0.49, 0.09	0.52, 0.08	0.134
**Change at week 3 (%)**			
∆SUV_max_	-33.7, 28.1	-35.3, 20.0	0.718
∆SUV_mean_	-18.4, 31.3	-24.3, 20.1	0.756
∆MTV	-45.1, 38.6	-53.4, 28.4	0.598
∆TLG	-54.2, 41.7	-64.7, 23.9	0.420
∆AUC-CSH	25.5, 18.4	20.9, 19.9	0.306

* Parameters compared using Mann-Whitney U test

### Correlation to locoregional recurrence free survival

Univariate Cox regression analysis of clinical features revealed that only alcohol intake and T stage were correlated to locoregional recurrence free survival, see supplementary Table 2. Univariate and multivariate Cox regression analysis was undertaken to correlate baseline and change in imaging features to locoregional recurrence free survival, see Table 3. There were no significant correlations between baseline imaging features including AUC-CSH and locoregional recurrence free survival. When comparing change in imaging features; ∆MTV (aHR 1.04; 95% CI 1.03–1.06; *p* < 0.001) and ∆TLG (aHR 1.05; 95% CI 1.05–1.06; *p* < 0.001) were correlated to locoregional recurrence free survival on multivariate analysis. Change in (∆) AUC-CSH (aHR 0.96; 95% CI 0.94–0.98; *p* = 0.004) was also correlated to locoregional recurrence free survival on multivariate analysis. Individual patient change in tumour heterogeneity (∆AUC-CSH) at week 3 during radiotherapy with corresponding locoregional recurrence status is presented as a waterfall plot in Fig. 2.

**Table 3:** Univariate and multivariate Cox regression analysis correlating week 0 and mid-treatment change in FDG-PET imaging features to locoregional recurrence free survival

Imaging feature	Univariate analysis			Multivariate analysis		
	HR	95% CI	*p* value	HR	95% CI	*p* value
**Week 0**						
SUV_max_	0.973	0.885–1.069	0.563			
SUV_mean_	0.869	0.693–1.090	0.225			
MTV	1.000	1.000–1.000	0.150			
TLG	1.001	0.999–1.004	0.272			
AUC-CSH	0.059	0.000-22.724	0.352			
**Change at week 3 (∆)**						
∆SUV_max_	1.007	0.990–1.024	0.417			
∆SUV_mean_	0.997	0.980–1.014	0.746			
∆MTV	1.031	1.020–1.043	**< 0.001**	1.041	1.026–1.056	**< 0.001**
∆TLG	1.013	1.006–1.021	**< 0.001**	1.045	1.027–1.063	**< 0.001**
∆AUC-CSH	0.960	0.934–0.986	**0.003**	0.961	0.936–0.988	**< 0.004**
- negative vs. positive	6.491	2.351–17.923	**< 0.001**	5.867	2.071–16.621	**< 0.001**

Metabolic tumour volume (MTV), Total lesional glycolysis (TLG)

Relative change in value (∆) = (Week 3– baseline)/baseline *100%

Hazard ratio (HR); 95% confidence interval (CI)

Multivariate analysis co-variates: p16 status, TNM stage, chemotherapy (Y/N)

**Fig. 3:**
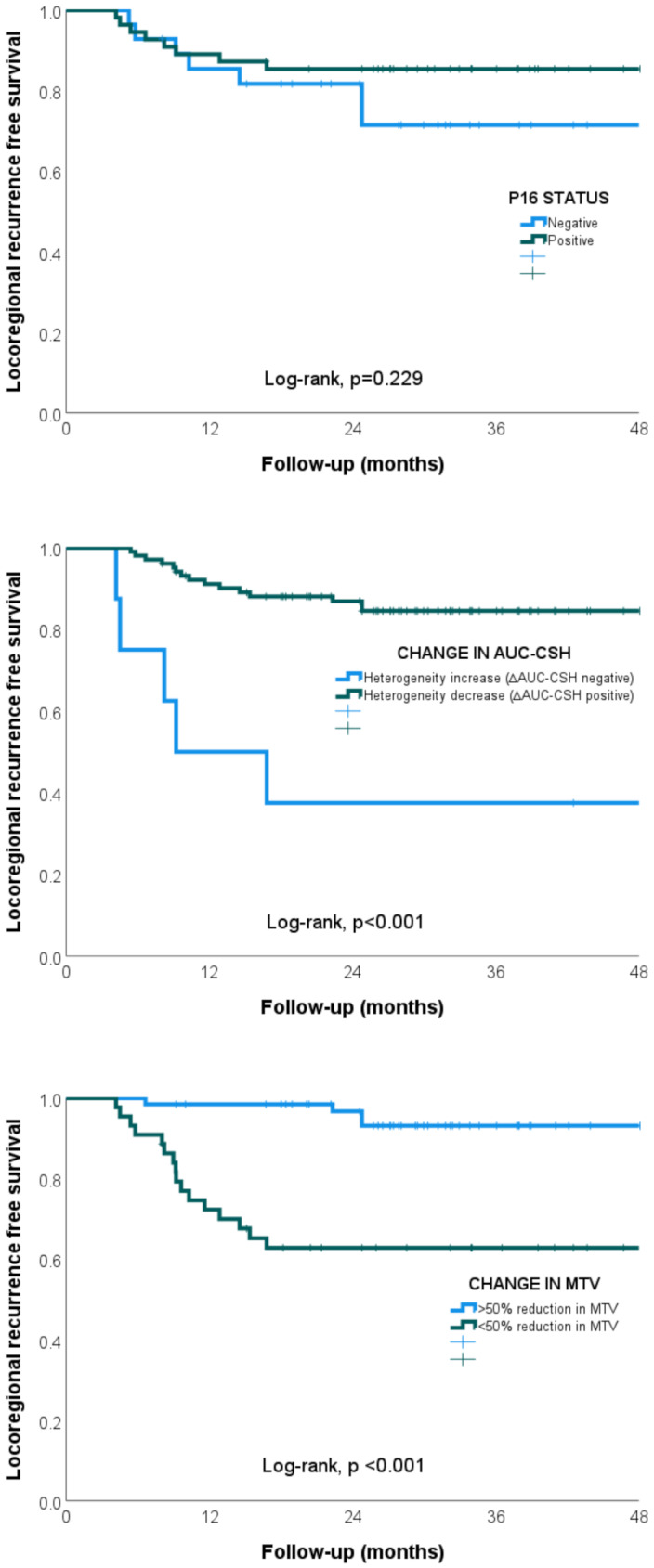
(**a-c**) Kaplan-Meier curve for locoregional recurrence stratified by response in primary tumour p16 status (**a**), change in tumour heterogeneity (> 0% ∆AUC-CSH) at week 3 (**b**), and change in metabolic tumour volume (≥ 50% ∆MTV) at week 3 (**c**)

Kaplan-Meier analysis was undertaken utilising pre-specified cut-off values for ∆AUC-CSH and ∆MTV. Using pre-specified cut-off value for ∆AUC-CSH of 0% revealed significant difference in locoregional recurrence at two years (2 year LRRFS 37.5% vs. 86.9%; log rank *p* < 0.001), see Fig. 3a. Using cut-off value for ∆MTV of 50% revealed significant difference in locoregional recurrence at two years (2 year LRRFS 62.8% vs. 96.7%; log rank *p* < 0.001), see Fig. 3b. No statistically significant difference in locoregional recurrence at two years was noted in our patient population between p16 positive and p16 negative patients (2 year LRRFS 85.4% vs. 79.1%, log rank *p* = 0.229), see Fig. 3c.

### Risk stratification using tumour heterogeneity

**Fig. 4:**
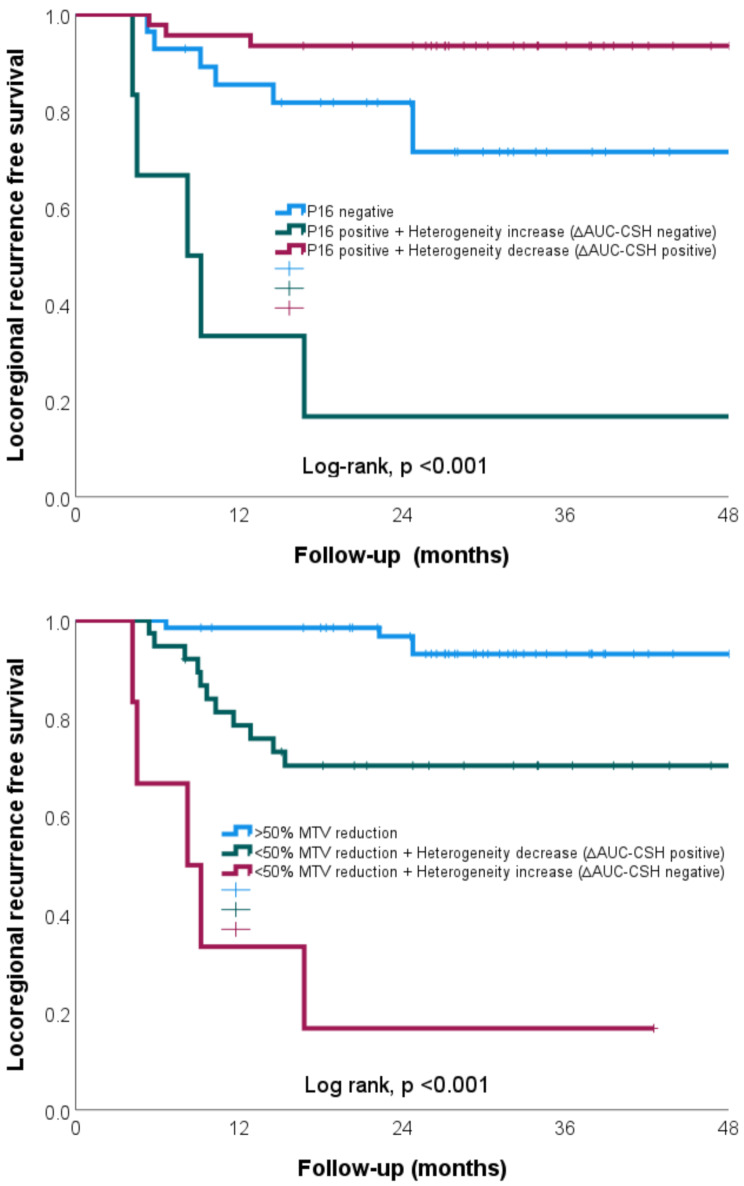
(**a-b**) Kaplan-Meier curve for locoregional recurrence based on 2 risk stratification methods; (a-b) by HPV association (p16 status) and change in tumour heterogeneity (> 0% ∆AUC-CSH) at week 3 and; (a-b) by response in change in metabolic tumour volume (≥ 50% ∆MTV) and tumour heterogeneity (> 0% ∆AUC-CSH) at week 3

To test the utility of combining tumour heterogeneity to clinical features, patients were stratified based on p16 status and pre-specified cut-off value of ∆AUC-CSH (0%) into three groups. There was a significant difference between p16 negative, p16 positive + unfavourable heterogeneity response (< 0% ∆AUC-CSH) and p16 positive + favourable heterogeneity response (> 0% ∆AUC-CSH) patients; 2 year LRRFS 79.1%, 16.7%, 93.5%, respectively, log rank *p* < 0.001), see Fig. 4a.

To test the utility of combining tumour heterogeneity and metabolic tumour volume, patients were stratified by pre-specified values of ∆AUC-CSH (0%) and ∆MTV (50%) into three groups. There was a significant difference between favourable volume response (≥ 50% ∆MTV), unfavourable volume response + favourable heterogeneity response (< 50% ∆MTV + > 0% ∆AUC-CSH) and unfavourable volume response + unfavourable heterogeneity response (< 50% ∆MTV + < 0% ∆AUC-CSH) patients; 2 year LRRFS 93.1%, 70.3%, 16.7%, respectively, log rank *p* < 0.001), see Fig. 4b.

## Discussion

Our study represents one of the largest mid-treatment FDG-PET imaging series in patients with oropharyngeal SCC treated with radiotherapy. We identified changes in tumour heterogeneity early during radiotherapy, which correlated to treatment outcomes. Additionally, we demonstrated that baseline and mid-treatment FDG-PET imaging markers were largely independent of p16 status. Moreover, by combining changes in tumour heterogeneity with either p16 status or changes in metabolic tumour volume, we were able to stratify patients into distinct risk groups based on their likelihood of recurrence. Based on our results, change in tumour heterogeneity measured using a simple, clinically implementable mid-treatment FDG-PET measure holds potential for tailored personalised treatment in the future.

We observed significant decrease in tumour heterogeneity early during the course of radiotherapy. Our study is the first to show that decrease in heterogeneity using ∆AUC-CSH, correlated to improved locoregional tumour control (2 year LRRFS 38% vs. 87%) in OPSCC. There is currently no published data correlating ∆AUC-CSH to clinical outcomes in head and neck malignancy. A study by Dong et al. containing 58 patients found that change in AUC-CSH in non-small cell lung cancer correlated to overall survival and progression free survival [34]. They found a correlation of ∆AUC-CSH using optimal cut-off values of 33% to OS and PFS. However, the mean increase in ∆AUC-CSH was much higher (45%) in the study by Dong et al. compared to our study population (24%). This variation can be explained by methodologically differences such as 50% non-SCC histology, SUV3.0 threshold for ROI delineation, post-processing of images and mid-treatment FDG-PET performed later during radiotherapy (week 4). A study by Mena et al. found a correlation between baseline FDG-PET-derived AUC-CSH and disease-free survival using an OC value of 0.64 (*p* = 0.04) in OPSCC patients [17]. In our population, using baseline AUC-CSH > 0.64 as a cut-off resulted in only five patients and there was no significant difference on Kaplan-Meier analysis (log rank *p* = 0.45). The baseline AUC-CSH values in study by Mena et al. were higher compared our study potentially due to differing post-processing, image reconstruction or filter application with resulting smoothing of the histogram data. In our dataset, the change in heterogeneity and not baseline heterogeneity correlated to locoregional recurrence. This finding suggests that changes in imaging features during treatment may serve as a better biomarker than a single baseline measure, which does not account for variation in individual tumour radiosensitivity. Our results could be explained by poorly responding tumours during radiotherapy harbouring radioresistant subclones resulting in a lower drop in SUV in that region, causing persistent heterogeneity and smaller increase in AUC-CSH at week 3 during radiotherapy.

FDG-PET is a readily available imaging modality that directly and indirectly measures functional aspects of the tumour, including tumour proliferation, altered metabolism and hypoxia, factors that are associated with radiotherapy resistance [5]. We demonstrated that changes in tumour heterogeneity measured using FDG-PET can successfully risk-stratify patients. For instance, patients with p16-positive and favourable heterogeneity changes (∆AUC-CSH) had a low rate of locoregional recurrence (2 year LRRFS 94%). Patients with good prognosis could be considered for de-escalation strategies such as radiotherapy dose de-escalation or alteration of concurrent systemic therapy. In contrast, patients with p16-positive tumours who had unfavourable heterogeneity (∆AUC-CSH) change during treatment did significantly worse, even when compared to p16-negative tumours (2 year LRRFS 17% vs. 79%, *p* < 0.001). Patients with poor prognosis could be considered for treatment escalation strategies such as radiotherapy dose escalation, adaptive radiotherapy dose painting, addition of hypoxia modifiers, or bail-out surgery [35–38]. A study by Wu et al. extracted a complex mid-treatment FDG-PET and CT imaging signature using tumour habitat features with an in-house developed software in OPSCC [39]. The imaging signature using a cut-off value containing a measure of tumour heterogeneity was correlated to PFS with a hazard ratio of 4.4 (*p* = 0.001) on multivariate Cox analysis. The current study using a pre-specified measure of increase in heterogeneity using a simple clinically implementable mid-treatment FDG-PET imaging histogram measure showed a strong correlation to LRR (HR 5.9, *p* < 0.001) on multivariate Cox analysis.

Using change in tumour heterogeneity may mitigate some of the limitations associated with currently utilised prognostic markers, such as p16 status. Despite the significant impact of p16 status on prognosis in OPSCC, it has failed to provide predictive ability in multiple risk adapted studies [1–4]. Potential reasons for the discordance is that a proportion of p16 positive tumours may not be HPV related and/or are inherently radioresistant. AUC-CSH may potentially identify these patients who would be unsuitable for treatment de-escalation strategies. Similarly, AUC-CSH may overcome a limitation of utilising mid-treatment FDG-PET metabolic tumour volume for response prediction. Treatment related inflammation resulting from radiotherapy can lead to falsely large metabolic tumour volume with relatively homogeneous uptake, especially when using a gradient or absolute threshold methods for tumour delineation [24].

HPV-associated and non-HPV associated OPSCC represents two distinct disease processes with different aetiology, demographics and prognosis [32]. There is also evidence of differing post-treatment FDG-PET imaging response assessment between the two processes [40]. When comparing changes in imaging features between HPV-associated and non-HPV-associated OPSCC, we found the only significant difference in baseline SUV_mean_ (6.8 vs. 5.9). We did not find a correlation between changes in imaging features, including tumour heterogeneity, by p16 status. Our data suggest that changes in FDG-PET imaging features could be used as a prognostic biomarkers independent of p16 status. To our knowledge, this study is one of the first to explore the relationship between HPV association and the changes in mid-treatment functional imaging features for tumour heterogeneity. When using baseline imaging only, there is currently limited and mixed evidence regarding the difference in functional imaging features between HPV associated and non-HPV-associated OPSCC [9]. A review by Lin et al. found that baseline FDG-PET imaging predicted treatment outcomes in non-HPV associated OPSCC, but not in HPV associated group on multivariate analysis [9]. Hence, the results of our study add considerably to the current literature on actionable biomarkers in HPV associated OPSCC.

Strengths of our study include its large cohort of a relative homogeneous patient population. Additionally, the mid-treatment FDG-PET imaging in our study was performed on the same scanner, resulting in a degree of self-normalisation and reducing image signal uncertainty. The repeat FDG-PET was performed at week 3 during radiotherapy, allowing for early assessment of treatment response and the opportunity to adapt treatment. In our study, the risk of bias were minimized by blinding the mid-treatment FDG-PET imaging results during treatment and concealing clinical outcomes during image analysis. However, our study was limited by its dual institutional nature. We utilised p16 staining as a surrogate for HPV association as per routine institutional policy and established clinical practice [41]. Our study could be strengthened by utilisation of central pathological review and use of HPV DNA/RNA testing. The discordance of p16 and HPV is likely to be very low in our patient population and hence unlikely to significantly impact the results of our study [42, 43]. The use of tumour heterogeneity for risk stratification utilised in our study is hypothesis-generating and requires further external validation prior to clinical implementation. We did not test other heterogeneity markers, such as higher-order radiomic features, which might have better correlation but also likely less reproducible with methodological variations [44]. We utilised AUC-CSH due to previous published literature, reproducibility, and ease of clinical implementation in future multi-institutional setting [15, 16].

## Conclusion

We identified changes in tumour heterogeneity early during radiotherapy that correlated with treatment outcomes in OPSCC and may help with response prediction. Based on our results, a change in tumour heterogeneity using a simple, clinically implementable mid-treatment FDG-PET measure has the potential to be used for tailored personalised treatment in the future.

## Electronic supplementary material

Below is the link to the electronic supplementary material.

Supplementary Material 1

## Data Availability

The datasets generated during and/or analysed during the current study are available from the corresponding author on reasonable request.
