# Supplementary material for: Mid-treatment changes in intra-tumoural metabolic heterogeneity correlate to outcomes in oropharyngeal squamous cell carcinoma patients
Source: EJNMMI Res. 2025 Apr 1;15:31. doi: 10.1186/s13550-025-01226-6 (PMC11961835; doi:10.1186/s13550-025-01226-6)

**Supplementary materials**

Supplementary table 1: Summary statistics of FDG-PET imaging features and their change during treatment

| **Parameter^a^** | **Before RT**  **(Week 0)** | **During RT**  **(Week 3)** | **Change from baseline (%)** | **P value** |
| --- | --- | --- | --- | --- |
| SUV_max_ | 13.5 ± 4.8 | 8.0 ± 3.3 | -37.5 ± 23.9 | **<0.001** |
| SUV_mean_ | 6.6 ± 2.0 | 4.8 ± 1.6 | -24.8 ± 26.2 | **<0.001** |
| MTV (cc) | 20.5 ± 22.0 | 10.3 ± 15.4 | -50.3 ± 31.3 | **<0.001** |
| TLG | 144.2 ± 166.5 | 48.8 ± 86.3 | -62.7 ± 28.3 | **<0.001** |
| AUC-CSH | 0.5 ± 0.1 | 0.6 ± 0.1 | 23.9 ± 19.7 | **<0.001** |

^a^Mean ± standard deviation

P values refer to Wilcoxon test

Metabolic tumour volume (MTV); Total lesional glycolysis (TLG)

Supplementary table 2: Univariate and multivariate Cox regression analysis correlating clinical features to locoregional recurrence free survival

|  | **Univariate analysis** | | |
| --- | --- | --- | --- |
|  | **HR** | **95% CI** | **p value** |
| **Clinical features** |  |  |  |
| Gender | 1.456 | 0.490-4.328 | 0.499 |
| Age | 1.018 | 0.973-1.064 | 0.442 |
| ECOG | 1.542 | 0.823-2.891 | 0.177 |
| Smoker (Y/N) | 2.337 | 0.688-7.942 | 0.174 |
| Smoking pack/year history | 1.010 | 0.993-1.026 | 0.261 |
| Alcohol intake | 1.399 | 1.021-1.919 | 0.037 |
| T stage | 1.935 | 1.143-3.274 | **0.014** |
| N stage | 0.935 | 0.687-1.271 | 0.666 |
| TNM stage (1/2/3/4) | 1.693 | 0.594-4.810 | 0.323 |
| Grade | 0.692 | 0.460-1.040 | 0.077 |
| P16 status | 0.847 | 0.462-1.551 | 0.591 |
| Chemotherapy (Y/N) | 26.057 | 0.154-4417 | 0.213 |

Relative change in value (∆) = (Week 3 – baseline)/baseline *100%

Hazard ratio (HR); 95% confidence interval (CI)

Supplementary table 3: Comparison of clinical features of patients by p16 status

|  | | P16 negative  (n=28) | P16 positive  (n=55) | P16 unknown  (n=31) | P value |
| --- | --- | --- | --- | --- | --- |
| Age at diagnosis (years) | | 63.1 ± 6.5 | 61.4 ± 10.1 | 62.0 ± 11.3 | 0.314 |
| Gender | Male | 21 (75%) | 49 (89%) | 27 (87%) | 0.219 |
|  | Female | 7 (25%) | 6 (11%) | 17 (15%) |  |
| Performance status | ECOG 0 | 11 (39%) | 37 (67%) | 15 (48%) | **0.035** |
|  | ECOG 1 | 14 (50%) | 17 (31%) | 42 (37%) |  |
|  | ECOG 2 | 3 (11%) | 1 (2%) | 9 (8%) |  |
| Smoker | No | 2 (7%) | 25 (46%) | 3 (10%) | **<0.001** |
|  | Yes | 26 (93%) | 30 (55%) | 28 (90%) |  |
| Smoking history (pack/yr) | | 38.9 ± 20.9 | 16.5 ± 21.4 | 34.3 ± 25.3 | **<0.001** |
| Alcohol intake | Nil | 3 (11%) | 16 (29%) | 5 (16%) | **0.003** |
|  | <1 SD/day | 5 (18%) | 18 (33%) | 5 (16%) |  |
|  | 1-3 SD/day | 5 (18%) | 8 (15%) | 7 (23%) |  |
|  | >3 SD/day | 8 (29%) | 8 (15%) | 9 (29%) |  |
|  | Ex-heavy (3 SD/day) | 7 (25%) | 5 (9%) | 5 (16%) |  |
| Primary tumour site | Tonsil | 12 (43%) | 28 (51%) | 11 (36%) | 0.120 |
|  | Base of tongue | 8 (29%) | 21 (38%) | 16 (52%) |  |
|  | Soft palate | 6 (21%) | 2 (4%) | 2 (7%) |  |
|  | PPW | 2 (7%) | 2 (4%) | 2 (7%) |  |
|  | UNPSCC | 0 (0%) | 2 (4%) | 0 (0%) |  |
| TNM stage | Stage 2 | 3 (11%) | 3 (6%) | 0 (0%) | **0.032** |
|  | Stage 3 | 7 (25%) | 5 (9%) | 6 (19%) |  |
|  | Stage 4a | 15 (54%) | 40 (73%) | 22 (71%) |  |
|  | Stage 4b | 3 (11%) | 5 (9%) | 2 (7%) |  |
|  | Stage 4c | 0 (0%) | 2 (4%) | 1 (3%) |  |
| Grade | Well differentiated | 5 (18%) | 4 (7%) | 3 (9%) | 0.947 |
|  | Mod differentiated | 5 (18%) | 8 (15%) | 4 (13%) |  |
|  | Poor differentiated | 7 (25%) | 29 (53%) | 1 (3%) |  |
|  | Unknown | 11 (39%) | 14 (26%) | 23 (74%) |  |
| Treatment | Radiotherapy alone | 4 (14%) | 8 (15%) | 5 (16%) | 0.975 |
|  | Chemoradiotherapy | 24 (86%) | 47 (86%) | 26 (84%) |  |

Continuous variables are presented in mean ± standard deviation and compared using Mann-Whitney U test. Categorical data are presented as numbers (%) and compared using Chi-square (x^2^) test or Fisher’s Exact test*.

Supplementary figures 1a-d: Scatter plot and corresponding Spearman correlation coefficient for tumour heterogeneity (AUC-CSH) and PET parameter values (SUV_max_, SUV_mean_, MTV, TLG) at baseline


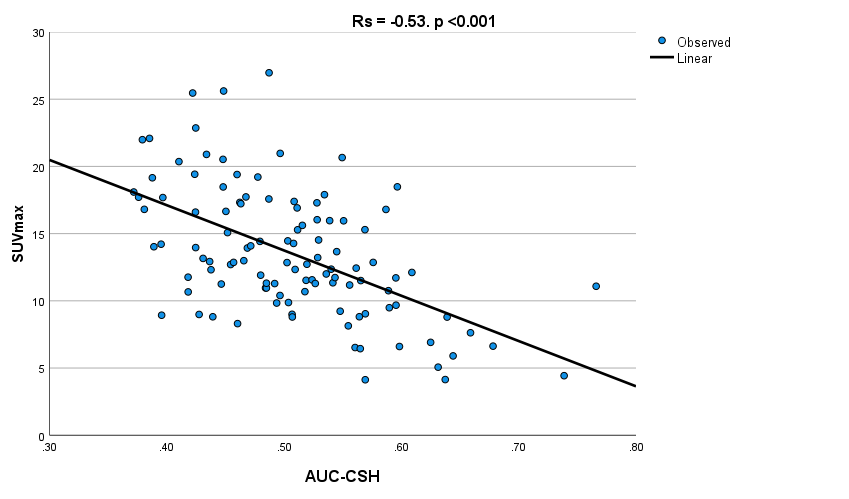

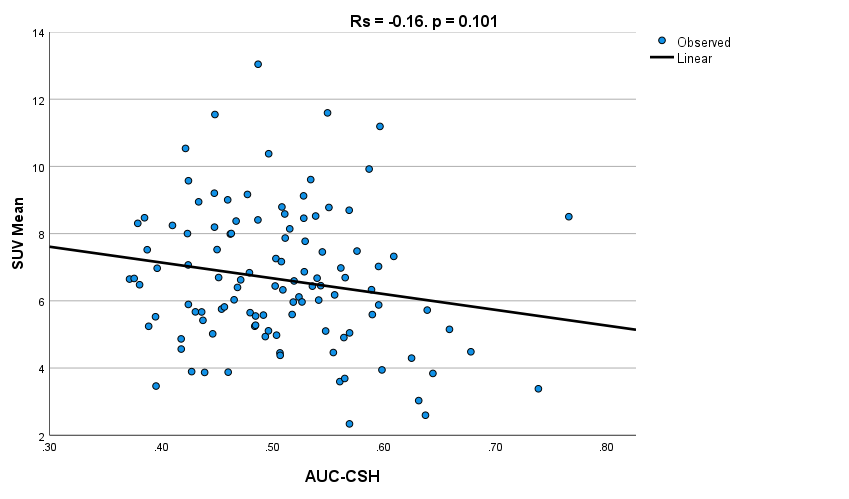

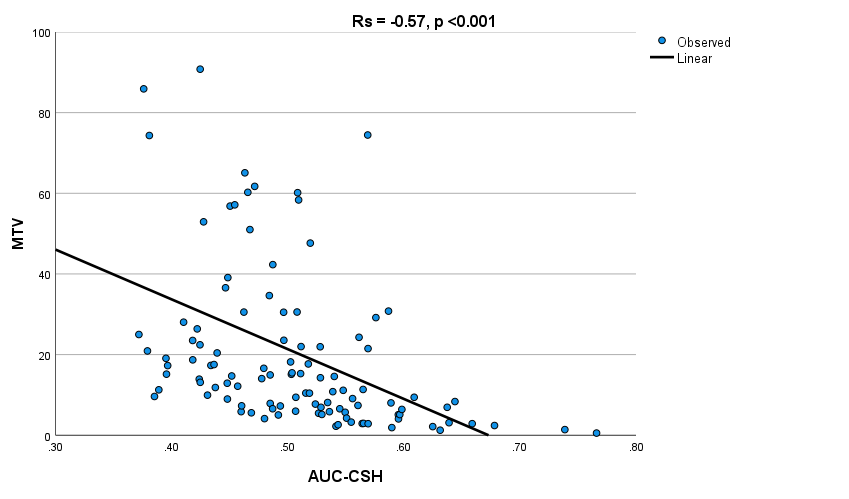

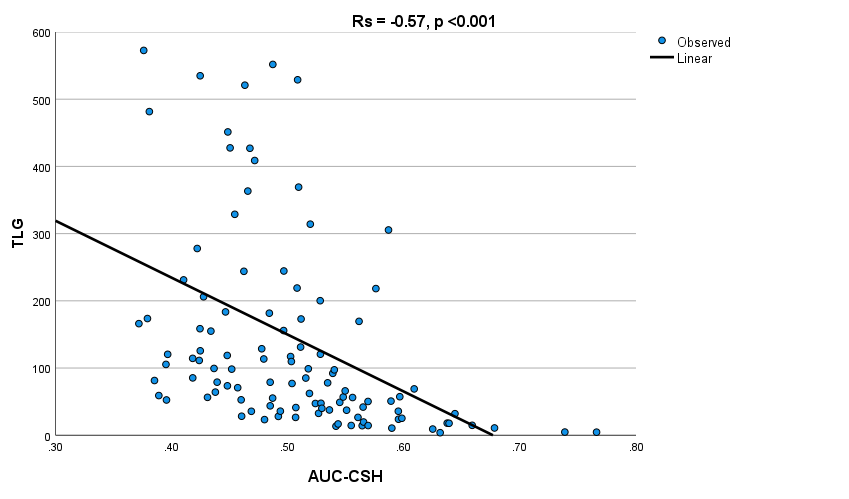


Supplementary figures 2a-d: Scatter plot and corresponding Spearman correlation coefficient for tumour heterogeneity (AUC-CSH) and PET parameter values (SUV_max_, SUV_mean_, MTV, TLG) at change at week 3 relative to baseline


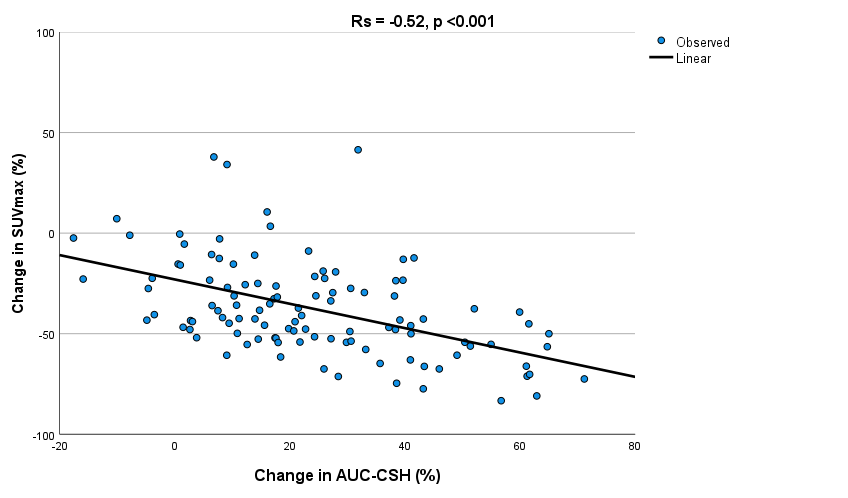

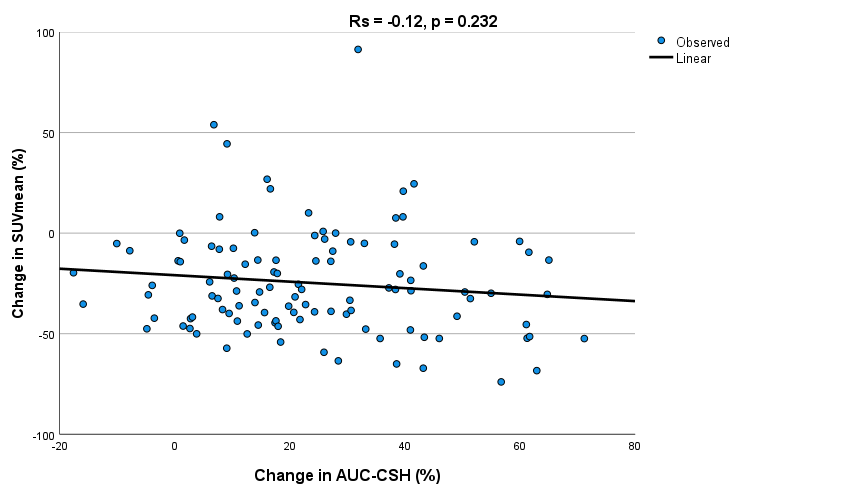


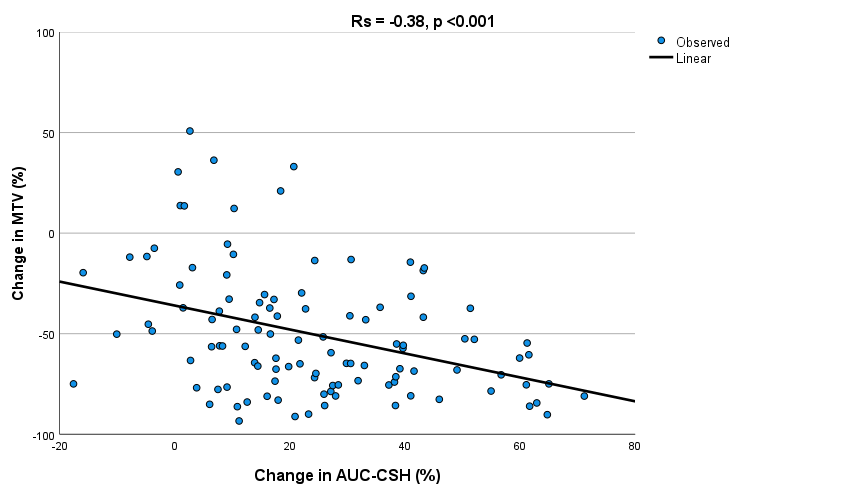

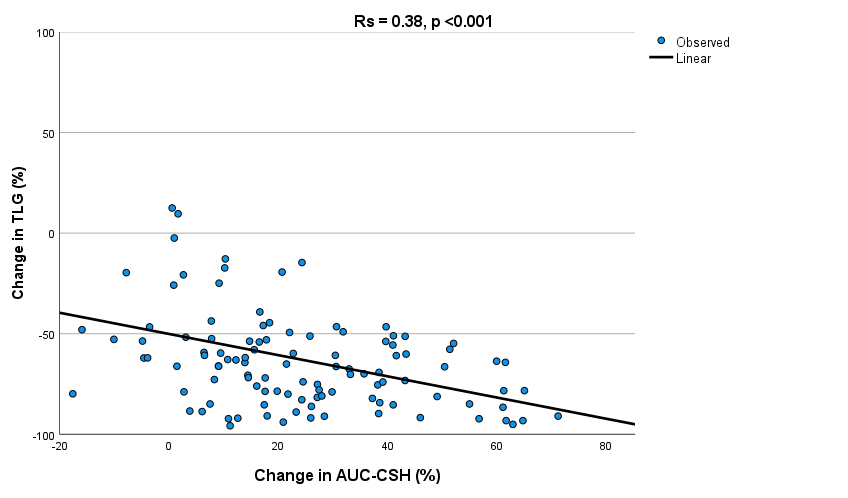

Supplement: Supplementary file 1 — Supplementary Material 1 [file 13550_2025_1226_MOESM1_ESM.docx]
